# NMR ^1^H, ^13^C, and ^15^N resonance assignments of the oncogenic Q61R variant of human NRAS in the active, GTP-bound conformation

**DOI:** 10.1007/s12104-025-10236-3

**Published:** 2025-05-02

**Authors:** Alok K. Sharma, Marco Tonelli, Marcin Dyba, William K. Gillette, Dominic Esposito, Dwight V. Nissley, Frank McCormick, Anna E. Maciag

**Affiliations:** 1 NCI RAS Initiative, Cancer Research Technology Program, Frederick National Laboratory for Cancer Research, Leidos Biomedical Research, Inc., 8560 Progress Drive, Post Office Box B, Frederick, MD 21701 USA; 2 Biochemistry Department, National Magnetic Resonance Facility at Madison, University of Wisconsin-Madison, Madison, WI 53706 USA; 3 Helen Diller Family Comprehensive Cancer Center, University of California, San Francisco, CA 94158 USA

**Keywords:** Melanoma, GTPase NRAS, Q61R mutation, GTP, HSQC, Secondary structure

## Abstract

NRAS^Q61R^ is a frequent mutation in melanoma. Hydrolysis of GTP by NRAS^Q61R^ is reported to be much slower than other KRAS and NRAS mutants. Recent structural biology efforts for KRAS and NRAS proteins have been limited to X-ray crystallography and therefore lack insight into the structure and dynamics of these proteins in solution. Here we report the ^1^H^N^, ^15^N, and ^13^C backbone and sidechain resonance assignments of the G-domain of oncogenic NRAS^Q61R^-GTP (MW 19.3 kDa; aa 1–169) using heteronuclear, multidimensional NMR spectroscopy. NRAS^Q61R^-GTP is a conformationally stable protein in solution. The ^1^H–^15^N correlation cross-peaks in a 2D ^1^H–^15^N HSQC spectrum collected after 48 h at 298 K remained intact and only minimal signs of peak-broadening were noted for select residues. High resolution NMR allowed unambiguous assignments of the ^1^H–^15^N correlation cross-peaks for all aa residues, except Y40, in addition to a significantly large number of aliphatic and aromatic sidechain resonances. NRAS^Q61R^-GTP exhibits canonical secondary structural elements in the 5 (five) α-helices, 6 (six) β-strands, and associated loop regions as predicted in TALOS-N and CSI. Order parameter (RCI-*S*^2^) values predicted by TALOS-N indicate that the NRAS^Q61R^-GTP switch (SW) regions and overall backbone are less flexible than observed in KRAS4b-GTP. The SW region rigidification was validated in heteronuclear NOE measurements. ^31^P NMR experiments indicate that the G-domain of NRAS^Q61R^-GTP is in a predominant state 2 (active) conformation.

**Supplementary Information:**

The online version contains supplementary material available at 10.1007/s12104-025-10236-3.

## Biological context

*RAS* is frequently mutated in cancer. *RAS (KRAS, HRAS,* and *NRAS*) genes encode small GTPase proteins which work as molecular switches cycling between their GTP-bound active state and the GDP-bound inactive state. In their active, GTP-bound state, RAS proteins interact and activate several downstream effectors including the mitogen-activated protein kinase (MAPK) and phosphatidylinositol 3-kinase (PI3K) pathways (Moore et al. 2020). In normal cells RAS proteins are tightly regulated by guanine nucleotide exchange factors (GEFs) promoting GDP dissociation and GTP binding, and GTPase-activating proteins (GAPs) that stimulate the intrinsic GTPase activity of RAS to turn off signaling by converting RAS to its signaling inactive GDP-bound form. Mutations in RAS lock the protein in the active GTP-bound conformation, activating downstream signaling pathways resulting in tumor cell growth. KRAS mutations are drivers of numerous cancer types, including pancreatic ductal adenocarcinoma (86%), colorectal cancer (41%), and lung adenocarcinoma (32%), and predominantly occur in codon 12. In contrast, NRAS is the predominant isoform mutated in cutaneous melanoma (Moore et al. 2020), and these mutations occur mostly in codon 61 (Cancer Genome Atlas Network 2015). Among NRAS Q61 mutations, the Q61R mutation occurs most frequently (~ 27%); other significant oncogenic mutations are Q61K, Q61L, and Q61H (Burd et al. 2014).

RAS is composed of two structural domains: a highly conserved N-terminal nucleotide binding G-domain, and a C-terminal Hyper Variable Region (HVR) that plays a role in the localization of RAS to the plasma membrane for protein function (Hobbs et al. 2016; Prior et al. 2012). All isoforms share sequence identity in the N-terminal part of the G-domain (residues 1–86), that includes regions responsible for nucleotide binding, GTPase activity, and effector interactions. Specifically, these functionally important regions include the P-loop (residues 10–17) that binds to nucleotide phosphate, Switch-I (SW-I, residues 25–40) and Switch-II (SW-II, residues 57–75) that bind to the different GEFs (Boriack-Sjodin et al. 1998) and effector proteins (Simanshu et al. 2017). The effector binding regions of RAS proteins are identical, and the effectors bind to all isoforms. However, differences between the biological properties of RAS isoforms have been described. For instance, one in vivo study demonstrated functional differences between mutant KRAS and NRAS; specifically, genetically engineered mice expressing KRAS^G12D^ and NRAS^G12D^ from endogenous loci exhibited clear phenotypic differences (Haigis et al. 2008). Another in vivo study points to the different oncogenic potential of NRAS mutations; notably, NRAS^Q61R^ is more efficient at promoting melanoma than NRAS^G12D^ in mice (Burd et al. 2014).

A significant number of studies have focused on structure and function of KRAS. However, studies focused on oncogenic NRAS are limited, and are primarily based on X-ray crystallography. Detailed atomistic insight into the solution structure as well as the conformational dynamics of NRAS^Q61R^-GTP is lacking. To fill this gap, we have initiated NMR-based studies to characterize the solution structure and dynamics of the G-domain of NRAS^Q61R^-GTP. Here we have applied high-resolution multidimensional, multinuclear solution NMR methods and performed backbone and sidechain atom assignments of NRAS^Q61R^ bound to the natural substrate GTP (named NRAS^Q61R^-GTP hereafter). Results from the characterization of the secondary structure, backbone dynamics of SW regions, and identification of the conformational state(s) adopted by NRAS^Q61R^-GTP are presented.

## Methods and experiments

### Protein expression and purification

Gateway Entry clones encoding the NRAS variants used in this work (Hs.NRAS4b(1–169) Q61R, T35A-Q61R, and T35S-Q61R) were synthesized by ATUM (Newark, CA) for optimal expression in *E. coli*. Entry clones were then subcloned into pDest-566 (Addgene #11517) to produce final expression clones of the form His6-MBP-tev-POI (MBP, maltose-binding protein; tev, tobacco etch virus protease cleavage site (ENLYFQG); POI, protein of interest) using the protocols outlined in Esposito et al. (2009). The T35S/Q61R variant was cloned for expression in insect cells in the same format: His6-MBP-tev-Hs.NRAS (1–169) T35S-Q61R. The Entry clone for this variant was transferred to a baculovirus expression vector containing an amino-terminal His6-MBP fusion pDest-636 (baculovirus, Addgene #159574) as per Esposito et al. The expression clone for RAF1 RBD (52–131) was described previously (Dharmaiah et al. 2019).

NRAS proteins (except for the T35S-Q61R variant, described below) were expressed in *Vibrio natriegens* following the protocols described previously (Smith et al. 2024) for non-isotopic and ^15^N incorporation (using 1.0 g/L uniformly ^15^N-labelled NH_4_Cl obtained from Cambridge Isotope Laboratories, Tewksbury, MA, USA). To produce ^13^C/^15^N-labelled NRAS (1–169) Q61R, uniformly ^13^C-labelled D-glucose (Cambridge Isotope Laboratories, Tewksbury, MA, USA) was added at 2.0 g/L to the ^15^N production protocol referenced above. NRAS variant T35S-Q61R was expressed in the insect cells using the methods as described previously (Snead et al. 2022). Expression of RAF1 RBD was achieved using the autoinduction protocol as described elsewhere (Taylor et al. 2017).

All proteins were purified as described for KRAS4b previously (Kopra et al. 2020; Smith et al. 2024), with the omission of MgCl_2_ in the purification of RAF1 RBD. Briefly, the expressed proteins of the form His_6_-MBP-tev-POI, were purified from clarified lysates by IMAC and treated with TEV protease to release the target proteins. The target proteins were separated from other components of the TEV protease reaction by a second round of IMAC. Proteins were subsequently purified by preparative gel-filtration chromatography in final buffer (20 mM HEPES, pH 7.3, 150 mM NaCl, 2 mM MgCl_2_ (omitted for RAF1 RBD), and 1 mM TCEP). Peaks containing the desired protein were pooled and stored at – 80 °C until further use. GTP loading of NRAS protein was achieved as detailed previously (Sharma et al. 2024). All purified proteins showed apparent purity of > 95% as detected by Coomassie Blue staining after SDS-PAGE and were analyzed by intact mass spectrometry (Frank et al. 2024) to be within ± 2 Daltons of the predicted MW. Isotopically labeled proteins were assessed to have > 98% isotope incorporation by intact mass spectrometry (data not shown). GTP loading of proteins was ascertained by HPLC and mass spectrometry. Sample concentration was measured using a NanoDrop One microvolume UV–Vis spectrophotometer (Thermo Fisher Scientific, MA, USA).

### NMR spectroscopy

Four samples of ^13^C/^15^N-labeled and one ^15^N-labeled were prepared for NRAS^Q61R^-GTP (each of 0.8 mM concentration) in a solvent composition of 93% H_2_O/7% D_2_O that contained 20 mM MES-d_13_ (pH 6.5; DLM 4363, CIL), 50 mM NaCl, 100 mM KCl, 1 mM TCEP-d_16_, 2 mM MgCl_2_. A 100 µM 2,2-dimethyl-2-silapentanesulfonic acid (DSS) was included as internal standard, and 0.02% (w/v) NaN_3_ was added to the sample to avoid any unwanted bacterial growth over time. NMR experiments were carried out on a Bruker Avance 700 MHz spectrometer equipped with a 5-mm TCI cryoprobe (*inhouse*) and on Bruker Avance III HD spectrometers of field strengths 900 MHz, 750 MHz, and 600 MHz equipped with 5-mm TCI (Z-axis gradient) cryoprobes (NMRFAM). All triple-resonance and 3D NMR data were collected at 298 K with gradient-selected sensitivity-enhanced pulse programs (Sattler et al. 1999) using nonuniform sampling (NUS) with sampling rates of approximately 30% (Hyberts et al. 2010). All NMR data were processed on an Intel PC workstation running CentOS 7 using NMRPipe/NMRDraw (Delaglio et al. 1995) and SMILE for NUS reconstruction (Ying et al. 2017). The ^1^H, ^13^C, and ^15^N chemical shifts were referenced to the internal standard DSS using IUPAC-IUB recommended protocols (http://www.bmrb.wisc.edu/ref_info/cshift.html). Spectra were visualized and analyzed using CCPNMR analysis (Vranken et al. 2005). Assignments were made manually.

Backbone resonance assignments were accomplished by analyzing 2D ^1^H–^15^N HSQC, and triple-resonance 3D HNCACB, CBCA(CO)NH, HNCA, HN(CO)CA, and HNCO spectra. Sidechain assignments were completed using 2D constant-time ^1^H–^13^C HSQC and 3D (H)CC(CO)NH, H(CCCO)NH, HC(C)H-TOCSY, HC(C)H-COSY, and CCH-TOCSY spectra. Backbone assignment data were also collected and analyzed using BEST pulse sequences from the standard Bruker library integrated with Avance III version. The ^1^H–^15^N sidechain assignments for Asn and Gln residues were assigned using 3D ^15^N-edited NOESY-HSQC dataset. The sidechain resonances for aromatic residues were assigned in ^1^H–^13^C-HSQC, (HB)CB(CGCD)HD, (HB)CB(CGCDCE)HE, HC(C)H-TOCSY-aro with the aid from 2D ^13^C constant time HSQC, enabling detection of only methylene protons of aromatic residues (Bax and Grzesiek 1993) and validated in ^13^C-edited NOESY-HSQC. After each dataset acquisition, protein stability was assessed by 2D ^1^H–^15^N HSQC spectrum. ^1^H–^15^N heteronuclear NOE (hetNOE) data were collected in an interleaved manner on a Bruker 600 MHz spectrometer. The hetNOE was determined as the ratio of spectral peak height recorded with ^1^H saturation (NOE, with a 3 s proton saturation applied throughout the recycle delay) to that recorded without ^1^H saturation (NONOE, using a 3 s recycle delay). Uncertainty in measurements was determined in duplicate datasets. Secondary structural elements were predicted from the backbone chemical shifts of ^1^H^N^, ^15^N, ^13^C^α^, ^13^C^β^, and ^13^C’ atoms using the confidence scores of numbers 8 or 9 in the program TALOS-N (Shen and Bax 2013).

^31^P NMR measurements were made using 0.8 mM samples of unlabeled NRAS^Q61R^-GTP and NRAS^T35S/Q61R^-GTP in absence or presence of equimolar RAF1 RBD in a solvent composition of 93% H_2_O/7% D_2_O in a physiological pH buffer of 20 mM HEPES (pH 7.3), 150 mM NaCl, 2 mM MgCl_2_, and 500 µM 2,2-dimethyl-2-silapentanesulfonic acid (DSS) as internal standard. Data were collected at 278 K on a Bruker 500 MHz spectrometer (a 202 MHz frequency for ^31^P nuclei) equipped with a 5 mm Prodigy broadband cryogenic probe using 70° flip angle pulses, 9000 scans with an interscan delay of 7 s. NMR data were processed and analyzed in Bruker Topspin4.1.4. Peak nomenclature was followed as described elsewhere (Spoerner et al. 2010; Sharma et al. 2024). Spectra were collected and processed in identical manner and data were referenced to DSS.

### Extent of assignments and data deposition

**Fig. 1:**
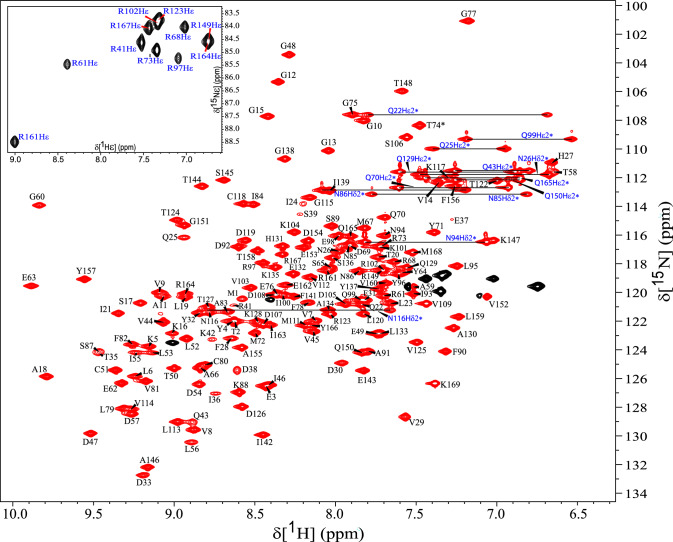
Two-dimensional ^1^H–^15^N HSQC spectrum shows amino acid (aa) residue assignments of NRAS^Q61R^-GTP. Data were collected on 700 MHz at 298K, pH 6.5. The assignments are annotated using the one letter aa code followed by its sequence number. Assignments for side-chain N–H correlations of Asn and Gln residues, connected by horizontal lines, are labeled. *Inset* shows Arg side-chain N–H correlations (black contours).

**Fig. 2:**
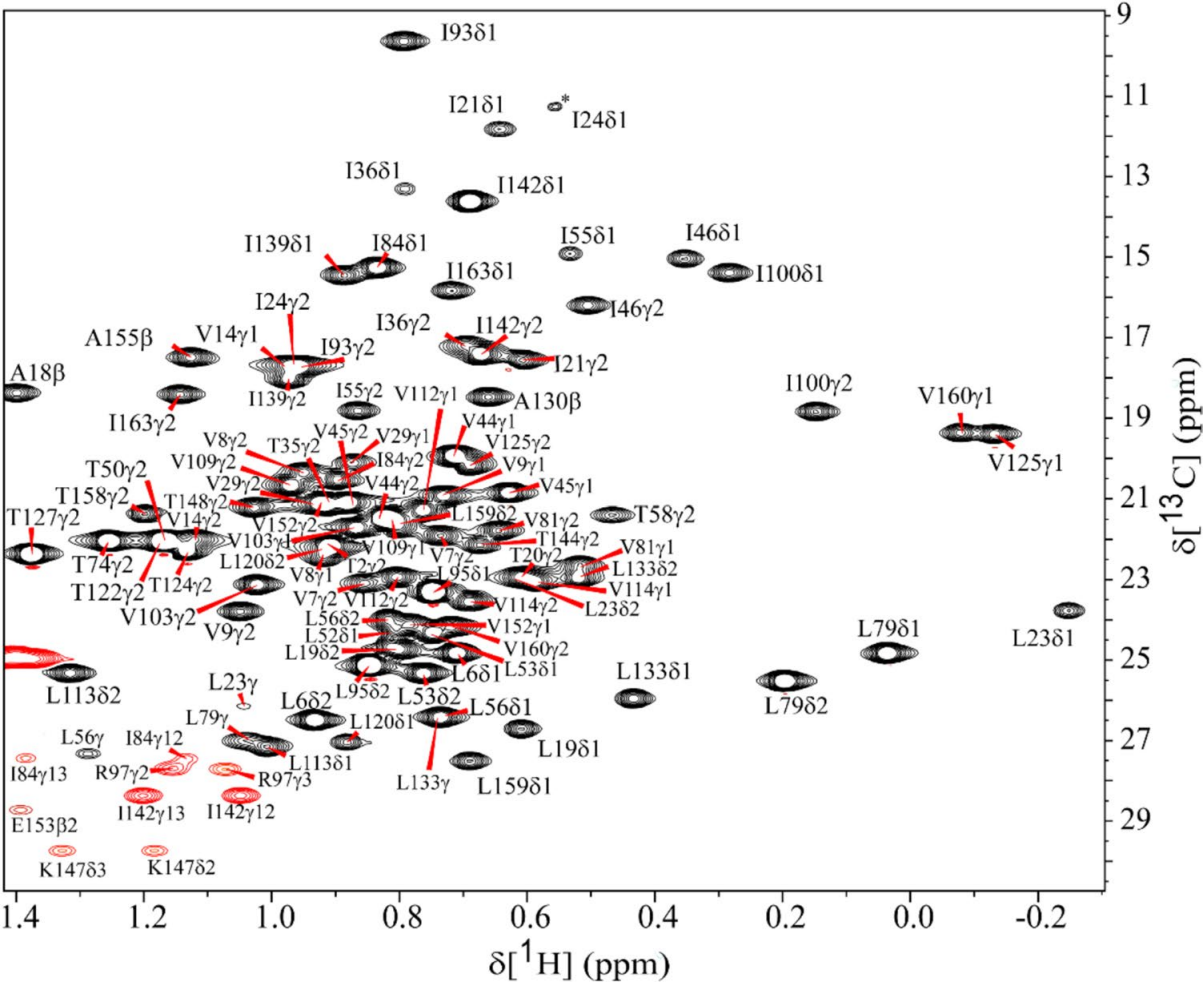
A zoomed view of annotated two-dimensional ^1^H–^13^C constant-time HSQC spectrum shows aa residue assignments for ^1^H–^13^C correlations of methyl groups belonging to Ile, Val, and Leu residues of NRAS^Q61R^-GTP. No stereospecific assignments were made. Peaks are assigned in CCPNMR v2.5. Data were collected on a Bruker 600 MHz at 298 K, pH 6.5. The assignments are annotated using the one letter aa code followed by its sequence number and atom labeling.

The ^1^H, ^13^C, and ^15^N chemical shifts assignments for NRAS^Q61R^-GTP have been deposited in BMRB (http://www.bmrb.wisc.edu/). Under the chosen solution conditions, the NMR sample remained stable at 298 K for 2–3 days with no additional peak emergence and extremely minimal peak degradation as noted in the 2D ^1^H–^15^N HSQC spectrum. Separate NMR samples were used each for triple-resonance experiments, TOCSY datasets, and NOESY suites of experiments. Good spectral dispersion of ^1^H–^15^N correlation cross-peaks in the 2D ^1^H–^15^N HSQC (Fig. 1) indicates that the protein adopts a well-folded conformation in solution. High-resolution NMR data allowed significant numbers of backbone and sidechain assignments. As noted in the 2D ^1^H–^15^N HSQC spectrum (Fig. 1), all ^1^H–^15^N backbone chemical shifts were assigned for 165 non-proline residues, except for Y40 (a total of 99.3% backbone amide correlations are assigned) for which signal appears to be broadened beyond detection. The carbonyl chemical shifts were assigned for all residues, except for D33 and S39 (98.8%). The ^1^H^ε^-^15^N^ε^ correlation assignments of all 11 Arg residues were made in se-^1^H–^15^N HSQC, HNCACB, and ^15^N-NOESY-HSQC spectra recorded using pulse programs tailored for observing Arg side chains (*inset*, Fig. 1). High-resolution spectral quality in the ^13^C dimension facilitated unambiguous assignments of the methyl groups of Ile, Leu, and Val residues (Fig. 2), as well as a large number of other ^1^H–^13^C correlation peaks of these and other residues. A significant number of ^13^C^α^ and ^1^H^α^ (98.8%; except for E37 and Y40) and ^13^C^β^ and ^1^H^β^ (99.4%; except for E37) atoms were assigned. Unambiguous assignments were made for ^1^H^δ^ and ^1^H^ε^ of aromatic rings for His and Tyr residues. The ^1^H^δ^, ^1^H^ε^, and ^1^H^ζ^ of most of the Phe residues were assigned, except for F82. Overall, the high-resolution HCCH-COSY, HCCH-TOCSY, and CCH-TOCSY data collected for aliphatic and aromatic regions, in conjunction with 3D NOESYs, made it possible to assign > 95% ^1^H atoms.

**Fig. 3:**
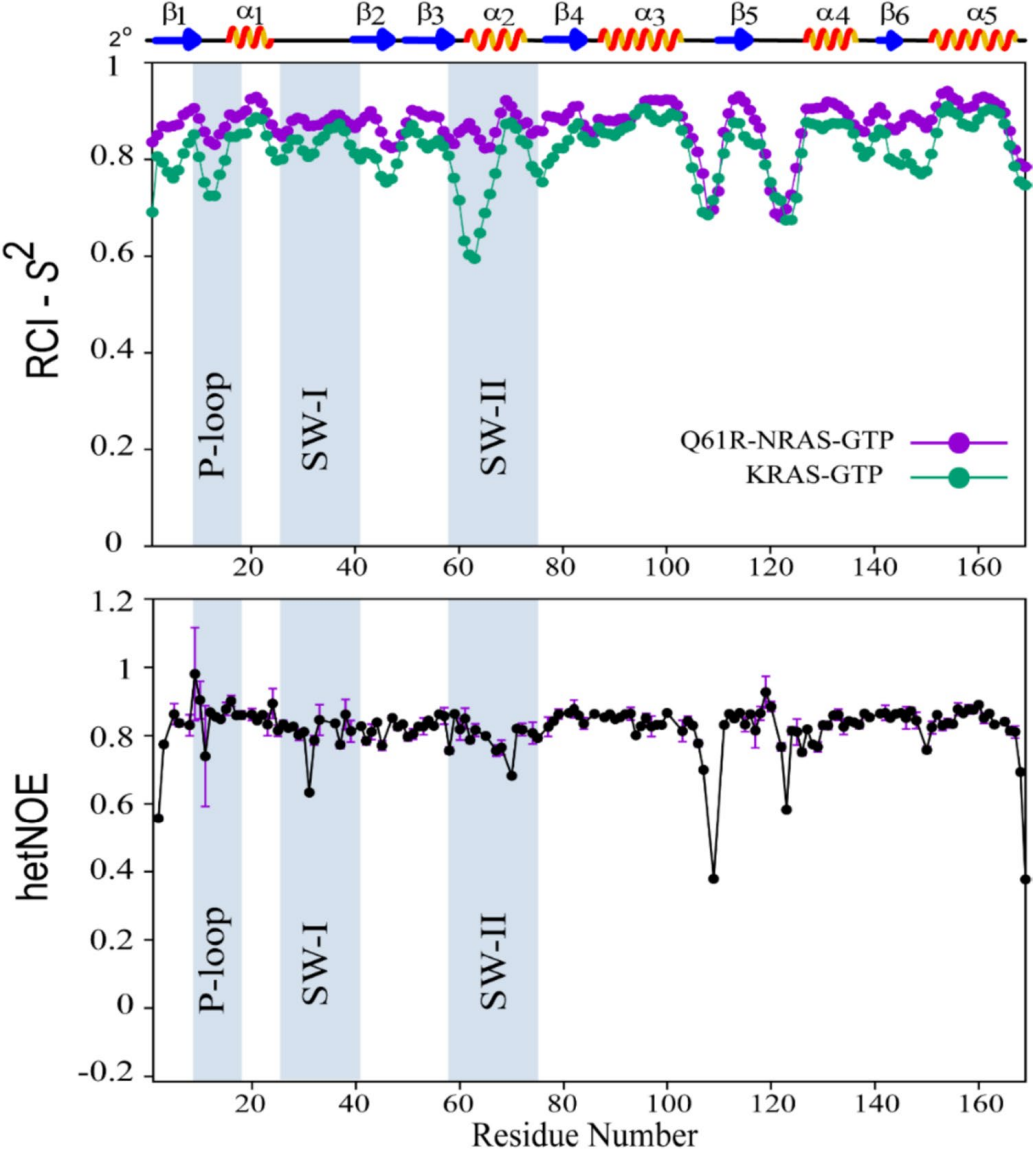
*Upper panel*: Predicted RCI-*S*^2^ order-parameter of amino acid residues of NRAS^Q61R^-GTP (in purple) and of KRAS4b-GTP (in green) as deduced from the assigned chemical shifts of ^1^H^N^ (our study also uses ^1^H^α^ chemical shifts), ^15^N, ^13^C^α^, ^13^C^β^, and ^13^C’ in TALOS-N are shown. AA regions encompassing P-loop, SW-I, and SW-II are highlighted in light blue background. Shown on top are the secondary structure (2°) elements (five α-helices, six β-strands, and associated loop regions) present in NRAS^Q61R^-GTP. *Lower panel*: The steady-state heteronuclear NOE values are plotted as a function of aa sequence. Error in NOE measurements (shown in purple) was determined from duplicate dataset.

Secondary structure of NRAS^Q61R^-GTP was determined from the ^1^H^N^, ^15^N, ^13^C^α^, ^13^C^β^, and ^13^C’ chemical shifts in programs TALOS-N (Shen and Bax 2013) and Chemical Shift Index (CSI) v 3.0 (Hafsa et al. 2015). Results show that the protein conformation of NRAS^Q61R^-GTP comprises a total of 11 secondary structural elements; namely, 5 α-helices and 6 β-strands are arranged in the order of β_1_-α_1_-β_2_-β_3_-α_2_-β_4_-α_3_-β_5_-α_4_-β_6_-α_5_. This observation generally matches closely to the canonical secondary structures present in NRAS conformations, or other RAS proteins (Gebregiworgis et al. 2024; Sharma et al. 2022). A consensus between high-confidence scores elicited in TALOS-N and those noted in CSI were used to deduce the residues encompassing the secondary elements that are β_1_ (T2-G10), α_1_(K16-I24), β_2_ (D38-I46), β_3_ (E49-D57), α_2_ (E62-T74), β_4_ (G77-A83), α_3_ (S87-K104), β_5_ (M111-N116), α_4_ (T127-S136), β_6_ (F141-E143), α_5_ (G151-M168). A plot showing the secondary structure probabilities obtained from TALOS-N as a function of aa sequence is presented (Supplementary Information Fig. S1). A noteworthy feature is the extended-length of helix α2 that includes aa 62–65 located at its N-terminus. The helical secondary structure of aa 62–65 is also seen in wildtype NRAS-GppNHp (PDB ID 5UHV), NRAS^Q61R−cmpd18^-GTP (PDB ID 6ZIZ), and NRAS^Q61K^-GTP (PDB ID 8VM2), but contrasts with the loop conformation noted in KRAS4b-GTP (Hansen et al. 2023). In GppNHp-bound KRAS4b crystal structures, these residues (aa 60–65) are also either absent or adopt flexible loop conformation. We used RCI based order parameter (*S*^2^) in TALOS-N for the chemical shift assignments of KRAS4b-GTP (BMRB ID 52021) (Hansen et al. 2023) and compared with the same in NRAS^Q61R^-GTP. These results, summarized in Fig. 3, identify relatively higher values of *S*^2^ of residues, and thus, less flexible backbone in NRAS^Q61R^-GTP than noted in wildtype KRAS4b. The two most flexible regions noted in NRAS^Q61R^-GTP fold are the loop regions between α_3_-β_5_ (L7: aa 105–110) and β_5_-α_4_ (L8: aa 119–125). Interestingly, GppNHp-bound KRAS4b conformational folds generally comprise a flexible SW-I region, however, in KRAS4b-GTP and NRAS^Q61R^-GTP this region is somewhat rigid (Fig. 3) and likely infers a role of non-natural GppNHp nucleotide on the protein dynamics (Menyhárd et al. 2020; Hansen et al. 2023; Sharma et al. 2024). Another interesting observation is that the SW-II region in wildtype KRAS4b is more flexible (dominated by lower *S*^2^ values in Fig. 3), whereas this region NRAS^Q61R^-GTP exhibits significant rigidity. This SW-II rigidity in helix α_2_ is partially attributed to the extended helical propensity observed at its N-terminal region. We characterized the dynamics of NRAS^Q61R^-GTP by determining the hetNOE values. Excluding the terminal residues, aside from loops L7 (encompassing aa D105-P110) and L8 (encompassing aa (K117-D126), none of the SW residues showed hetNOE < 0.6 (Fig. 3). The hetNOE data validates a decreased flexibility of SW residues and corroborates the trend observed in chemical-shift based *S*^2^ values. Overall, these data indicate that the conformational fold of NRAS^Q61R^-GTP in solution is relatively more compact and less dynamic in the SW regions compared to wildtype KRAS4b. A future study of three-dimensional solution structures and dynamic parameters of NRAS^Q61R^-GTP will provide more details.

**Fig. 4:**
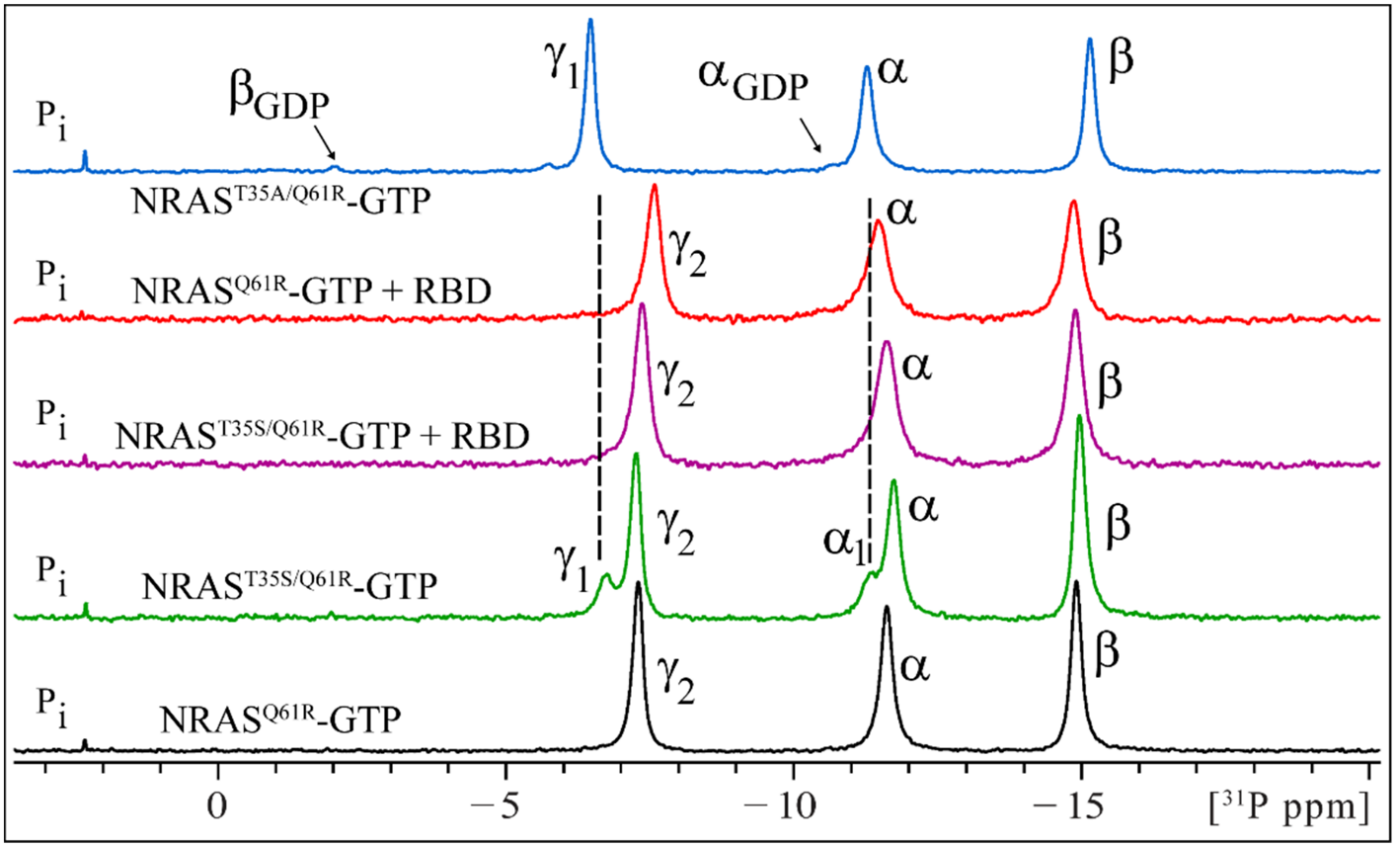
One-dimensional ^31^P NMR spectra of NRAS^Q61R^-GTP and of NRAS^T35S/Q61R^-GTP in absence and presence of effector RAF1 RBD at 278 K. At the *bottom* is shown the spectrum for NRAS^Q61R^-GTP. The α_1_, β_1_ and γ_1_ peaks represent state 1, and α_2_, β_2_ and γ_2_ peaks the state 2 conformation of the protein; peak nomenclature follows the pattern as noted elsewhere (Spoerner et al. 2010) and described recently (Sharma et al. 2024). The NRAS^T35S/Q61R^-GTP spectrum shows a conformation equilibrium between state 1 (downfield-shifted γ_1_ peak) and state 2 (γ_2_ peak). Comparison of these two spectra demonstrate that the γ peak noted in the NRAS^Q61R^-GTP spectrum belongs to γ_2_ (state 2) conformation. The state 1 population (γ_1_ peak) noted in NRAS^T35S/Q61R^-GTP spectrum disappears in presence of RAF1 RBD protein, and only a state 2 representing γ_2_ peak is noted, as expected. Shown on *top* is the spectrum for T35A mutant of NRAS^Q61R^-GTP protein that represents a state 1 only conformation, and validates the notion that NRASQ^61R^-GTP adopts a state 2 conformation in solution. No peak perturbation is noted for NRAS^Q61R^-GTP in presence of RAF1 RBD

GTP-bound RAS proteins generally exist in an equilibrium between two conformational states, state 1 (inactive) and state 2 (active) mainly represented by γ_1_ and γ_2_ peaks of γP atom from RAS bound nucleotide, respectively. These peaks can be detected in the ^31^P NMR spectrum (Spoerner et al. 2010; Sharma et al. 2024). GppNHp-bound NRAS^Q61R^ was reported to only adopt the state 2 conformation (Rennella et al. 2024). Here we extend the understanding of the conformational equilibria for NRAS^Q61R^ bound to its natural substrate GTP. The ^31^P spectrum of NRAS^Q61R^-GTP (Fig. 4) shows three canonical peaks belonging to the α-, β-, and γ- phosphate of GTP. A comparison of this spectrum with that of T35S counterpart of NRAS^Q61R^-GTP (*i.e.* NRAS^T35S/Q61R^-GTP), demonstrates that the γP peak in NRAS^Q61R^-GTP represents the state 2 (γ_2_)-only conformation. Incorporation of the T35S mutation is known to shift the conformational equilibrium towards state 1 (Spoerner et al. 2010; Sharma et al. 2024). As noted in Fig. 4, emergence of a downfield shifted peak γ_1_ is only noted in the spectrum of NRAS^T35S/Q61R^-GTP and not in NRAS^Q61R^-GTP. In the presence of equimolar effector protein RAF1-RBD, the conformational equilibrium of NRAS^T35S/Q61R^-GTP shifts in favor of the state 2 conformation (Fig. 4). The presence of RAF1-RBD does not cause any changes to the singly observed γ_2_ peak of NRAS^Q61R^-GTP.

Observation of all the SW residues of KRAS4b-GTP in a 2D ^1^H–^15^N HSQC spectrum at 298 K is generally a challenging task, presumably due to their involvement in the dynamics between the conformationally heterogeneous state 1 and state 2 conformations (Menyhard et al. 2020). Assistance from NMR measurements at lower temperature was required to unambiguously assign these SW residue signals in the 2D ^1^H–^15^N HSQC spectrum (Hansen et al. 2023). However, all the SW residues (except Y40) of NRAS^Q61R^-GTP are detected in its 2D ^1^H–^15^N HSQC spectrum at 298 K, most likely due to significant decrease in their conformational mobility. Extremely slow intrinsic hydrolysis rates and the presence of a predominant state 2 conformation are likely factors that contribute to the reduced flexibility of SW residues in NRAS^Q61R^-GTP compared to KRAS4b-GTP. A future NMR study directly comparing KRAS4b^Q61R^-GTP with NRAS^Q61R^-GTP would provide clues to the role of the Q61R mutation on their conformational dynamics.

In conclusion, we have provided the NMR assignments of NRAS^Q61R^-GTP in the state 2 conformation. The data are useful for future NRAS structural studies, for example, in determining the impact of codon 61 mutations by comparing the spectral properties of Q61R with other stalled nucleotide-hydrolysis NRAS oncogenic mutants Q61H and Q61K. Additionally, our data will be useful in binding site mapping experiments with small molecule inhibitors in drug discovery campaigns.

## Supplementary Information

Below is the link to the electronic supplementary material.

Supplementary file1 (DOCX 356 kb)

## Data Availability

^1^H, ^13^C, and ^15^N chemical shift assignments for GTPase NRAS^Q61R^-GTP were deposited in the Biological Magnetic Resonance Bank (BMRB) with accession code 52910.
