# Supplementary material for: NMR 1H, 13C, and 15N resonance assignments of the oncogenic Q61R variant of human NRAS in the active, GTP-bound conformation
Source: Biomol NMR Assign. 2025 May 2;19(1):195–203. doi: 10.1007/s12104-025-10236-3 (PMC12116848; doi:10.1007/s12104-025-10236-3)
Supplement: Supplementary file 1 — Supplementary file1 (DOCX 356 kb) [file 12104_2025_10236_MOESM1_ESM.docx]

Supplementary Information (SI)

**NMR ^1^H, ^13^C, and ^15^N resonance assignments of the oncogenic Q61R variant of human NRAS in the active, GTP-bound conformation**

Alok K Sharma^1^, Marco Tonelli^2^, Marcin Dyba^1^, [William K Gillette](https://pubmed.ncbi.nlm.nih.gov/?term=Gillette+WK&cauthor_id=34686998)^1^, Dominic Esposito^1^, Dwight V Nissley^1^, Frank McCormick^1,3^, and Anna E Maciag^1^.

^1^NCI RAS Initiative, Cancer Research Technology Program, Frederick National Laboratory for Cancer Research, Leidos Biomedical Research, Inc., Frederick, MD, 21701, USA. ^2^National Magnetic Resonance Facility at Madison, Biochemistry Department, University of Wisconsin-Madison, Madison, WI, 53706, USA. ^3^Helen Diller Family Comprehensive Cancer Center, University of California, San Francisco, CA, 94158, USA.

Corresponding Author:

Alok K. Sharma, Ph. D.

Cancer Research Technology Program

Leidos Biomedical Research, Inc.,

Frederick National Laboratory for Cancer Research

8560 Progress Drive,

Frederick, MD 21701, USA

Tel: 301-846-6402

Mailing address: Post Office Box B, Frederick, MD 21702

Email: alok.sharma@nih.gov

**
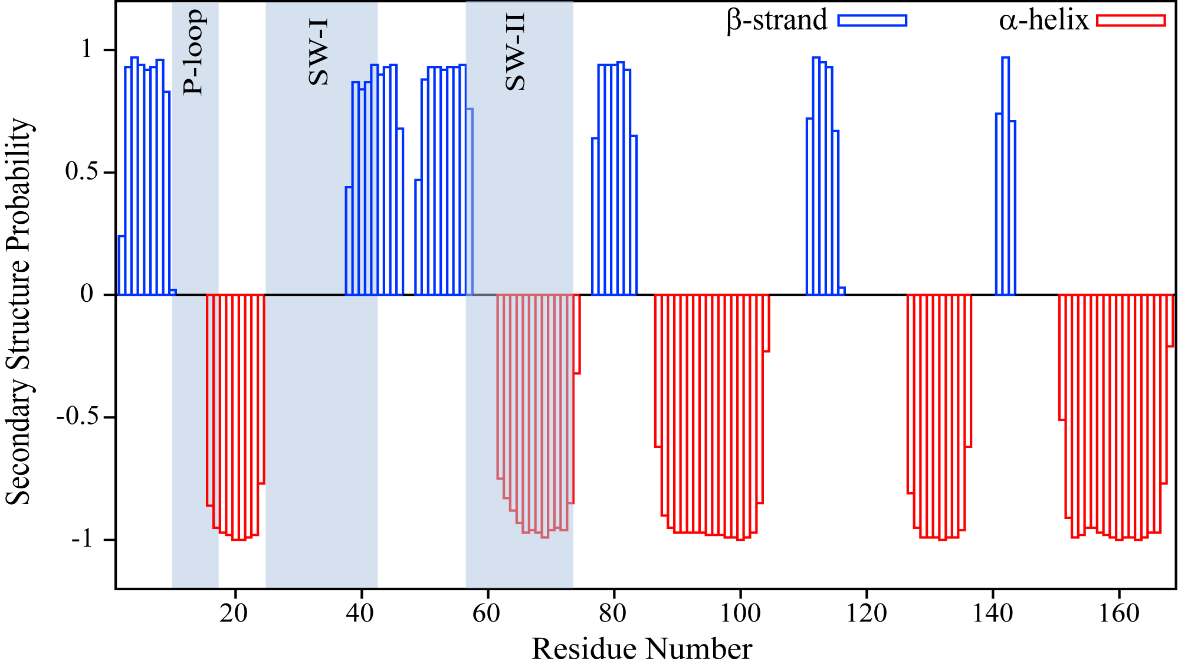
**

**Fig. S1** A plot showing the secondary structure probability (SSP) *vs* aa residue number of NRAS^Q61R^-GTP determined from TALOS-N. SSP scores closer to -1, 0, and 1 represent higher propensity α-helices (red bars), loop regions, and elongated β-strands (blue bars), respectively. AA regions encompassing P-loop, SW-I, and SW-II are highlighted in light blue background.
